# Analysis of Mechanical Properties and Permeability of Trabecular-Like Porous Scaffold by Additive Manufacturing

**DOI:** 10.3389/fbioe.2021.779854

**Published:** 2021-12-21

**Authors:** Long Chao, Chen Jiao, Huixin Liang, Deqiao Xie, Lida Shen, Zhidong Liu

**Affiliations:** ^1^ College of Mechanical and Electrical Engineering, Nanjing University of Aeronautics and Astronautics, Nanjing, China; ^2^ State Key Laboratory of Pharmaceutical Biotechnology, Division of Sports Medicine and Adult Reconstructive Surgery, Department of Orthopedic Surgery, Nanjing Drum Tower Hospital, The Affiliated Hospital of Nanjing University Medical School, Nanjing, China; ^3^ Jiangsu Engineering Research Center for 3D Bioprinting, Nanjing, China

**Keywords:** additive manufacturing, voronoi tessellation, permeability, mechanical properties, stress shielding, bone scaffold, bionic structure

## Abstract

Human bone cells live in a complex environment, and the biomimetic design of porous structures attached to implants is in high demand. Porous structures based on Voronoi tessellation with biomimetic potential are gradually used in bone repair scaffolds. In this study, the mechanical properties and permeability of trabecular-like porous scaffolds with different porosity levels and average apertures were analyzed. The mechanical properties of bone-implant scaffolds were evaluated using finite element analysis and a mechanical compression experiment, and the permeability was studied by computational fluid dynamics. Finally, the attachment of cells was observed by confocal fluorescence microscope. The results show that the performance of porous structures can be controlled by the initial design of the microstructure and tissue morphology. A good structural design can accurately match the performance of the natural bone. The study of mechanical properties and permeability of the porous structure can help address several problems, including stress shielding and bone ingrowth in existing biomimetic bone structures, and will also promotes cell adhesion, migration, and eventual new bone attachment.

## 1 Introduction

In China, 15 million patients with bone defects need artificial bone tissue each year (Attar et al., 2018). However, the porous structure of common bone scaffolds involves various problems (Carla et al., 2018; Zhao et al., 2019), such as single morphology and insufficient osteogenesis ability. Porous scaffolds play an important role in the proliferation and differentiation of human cells. As structures for cell growth, scaffolds require a roughness surface to facilitate the attachment and proliferation of cells on the scaffolds. Moreover, porous scaffolds play an important role in nutrient transport and waste removal during cell growth. The ideal porous structure possesses bone characteristics (i.e., microstructure geometric features and mechanical, biological, and nutrient transport) to realize similar degrees of cell infiltration and diffusion (Chang et al., 2020; Falkowska et al., 2020; Ma et al., 2020; Wang et al., 2020). Trabecular-likes consist of a large number of intertwined trabecular bones and match the internal bones of humans. Trabecular bones are arranged in the same direction as that of bone stress and tension and thus can bear larger weights. It provides a template for constructing artificial bone scaffolds (Feng et al., 2017; Alias and Buenzli, 2018; Zhang et al., 2019).

Advances in medical three-dimensional printing have led to opportunities for creating complex artificial bone-implant structures (Kantaros et al., 2015; Melancon et al., 2017). Computer-aided design tools have been used to mimic scaffolding structures close to real human bone tissue. A porous structure design can be divided into rules and irregular structure, rules of porous structure modeling methods, including the unit method, the topological optimization method, and the three-cycle minimum surface method (Kumar et al., 2017; Wang et al., 2019; Rana et al., 2021). The rules of porous structures show poor biomechanics and liquidity; in addition, because small changes in the unit cell in the rules of the porous structure can lead to the internal structure of the overall change (Chen et al., 2017; Li et al., 2017; Nguyen-Van et al., 2020). Thus, the local distribution of the shape and aperture is difficult to control. Inverse modeling based on computed tomography/magnetic resonance imaging can also accurately simulate human bone tissue and prove the advantage of an irregular porous structure. However, the porous model obtained by reverse modeling is difficult to modify in the late stages (Liu et al., 2016; Ibrahim et al., 2018; Maskery et al., 2018; Onal et al., 2018; Lu et al., 2019; Ouyang et al., 2019; Samoilenko et al., 2019).

**FIGURE 1:**
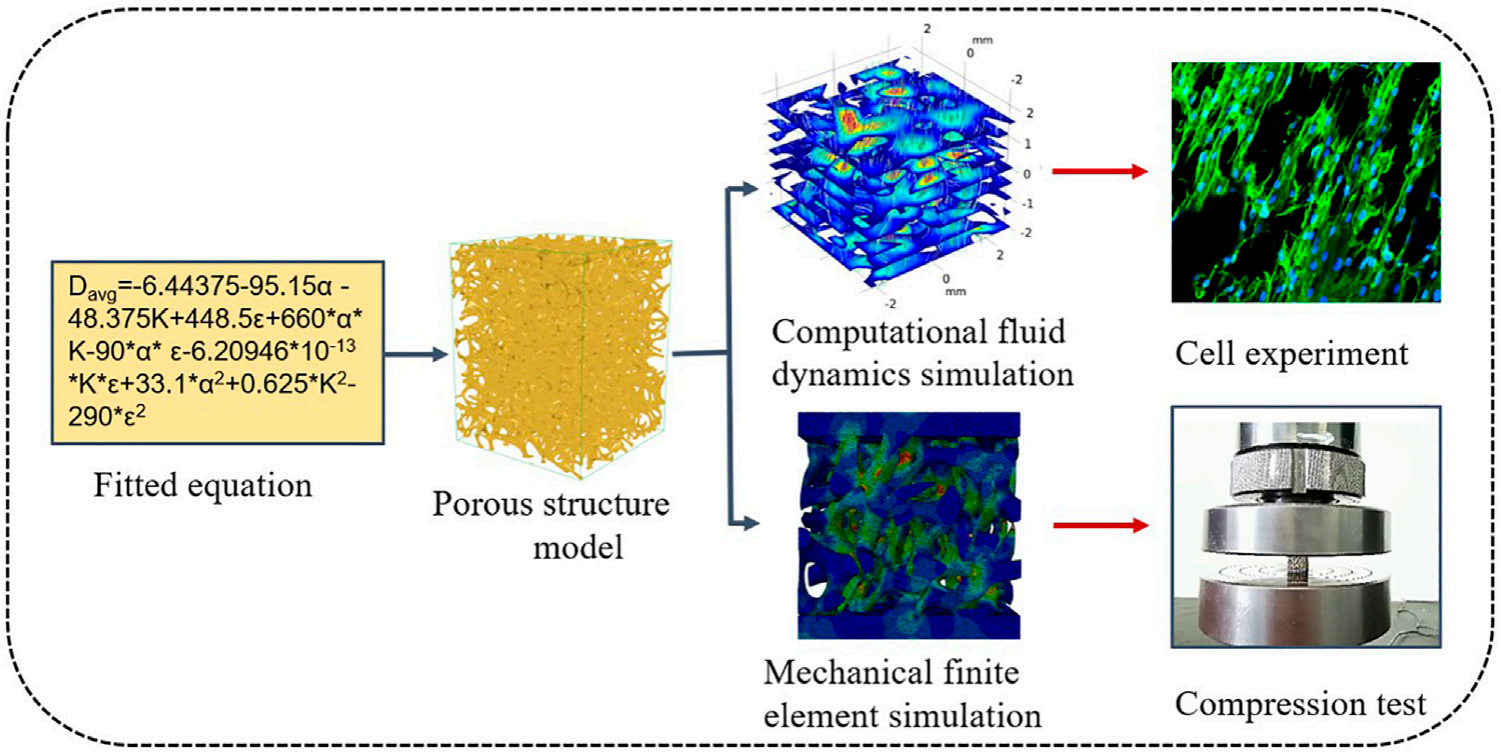
Research process.

Porous structure modeling based on Voronoi tessellation has increasingly gained interest in recent years (Liang et al., 2019; Du et al., 2020; Lei et al., 2020). The structure has a large aperture distribution range because of the similarity between the designed porous structure and the complex microstructure of human bones, thus addressing the problem concerning the small aperture distribution range of regular structures. Meanwhile, by adjusting the parametric design of structures based on Voronoi tessellation, the requirements set for the mechanical properties and permeability of different porous structures are met. Currently, porous structure modeling based on Voronoi tessellation is limited by a specific modeling technique and cannot easily control the aperture and porous structure. In the current study, Voronoi tessellation is applied in the design of spongy porous structures. The design parameters of the porous structure are determined, including porosity, aperture distribution, and the relationship between mechanical properties. The porosity and average aperture of porous structures are regulated by controlling the design parameters, combined with finite element analysis, to show the applicability of the method. This study belongs to the 3D-printed Biomaterials in Osteochondral Repair. The irregular porous structure constructed by the Voronoi tessellation, porous structure is suitable for the growth of bone tissue due to its large and small pores of different shapes. the mechanical and permeability properties of trabecular-like porous scaffolds with different porosity and average apertures were analyzed, The results of the analysis are verified using a compression test and biological cell culture experiments. The research of mechanical properties and permeability of the porous structure can help address several problems, including stress shielding and bone ingrowth in existing biomimetic bone structures. The research process is shown in Figure 1.

## 2 Design and Methods

### 2.1 Design of Porous Structures

We propose a controllable irregular porous structure method based on probability balls and the Voronoi–Tessellation approach (Du et al., 2020). The software Grasshopper is used to control the irregularity of the lattice via the probability ball for design. The basic idea is to generate a regular lattice with a certain distance in the space and establish a spherical region with the regular point as the center and randomly generate a seed point in each spherical region. In accordance with the Voronoi–Tessellation principle, the random seed lattice is connected, generating a Voronoi three-dimensional framework. The characteristic parameters of irregular porous structures mainly include the average aperture (D), porosity (*Φ*), irregularity coefficient (*ε*), point spacing (a), probability sphere radius (R), and aperture coefficient (K). K is the ratio of the pore area Spi to the corresponding cellular surface area Sci—that is, K = Sci/Spi.

The two factors of point spacing and irregularity coefficient only slightly affect porosity, and the porosity mainly depends on the pore size, exhibiting a strong linear relationship. The structure we designed is irregular porous structure, pore size distributed within a definite range, it is not a single value (Liang et al., 2019), so we use average aperture. To obtain a representative average aperture and a porous structure with good morphological bionic characteristics, an irregularity coefficient ranging from 0.4 to 0.5 was selected. The suitable aperture range for bone cell ingrowth is 200–1,200 μm. With this range considered, the interval of point spacing was set to 1.5–2.5 μm. Finally, the aperture coefficient range was set to 0.5–0.9, given that the porosity of a trabecular-like ranges from 50 to 90%. We constructed the fitting function relation (Eq. 1) to depict the relationship between porosity and average aperture and design parameters, as follows:

$$\begin{array}{l} D_{avg}=-6.44375-95.15a-48.375K+448.5\varepsilon \\ +660\times a\times K-90\times a\times \varepsilon -6.20946\times 10^{-13}\times K\times \varepsilon \\ +33.1\times a^{2}+0.625\times K^{2}-290\times \varepsilon^{2} \end{array}$$ (1)

**FIGURE 2:**
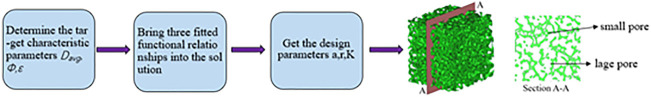
Porous structure design process.

The fitted equation can be used to calculate the design parameters depending on the target average aperture. The effects of average aperture, porosity, and pore structure on the mechanical and permeability properties of porous metals were evaluated in this study. Bone integration and bone ingrowth can be facilitated and the advantages of a porous structure can be fully utilized only when the aperture is within a reasonable range. Therefore, to provide adequate space and sufficient mechanical support for cell diffusion, the aperture of the porous scaffold should be controlled as much as possible. In the design of an irregular porous mechanism based on Voronoi tessellation, irregular porous structures with different apertures were modeled under similar conditions to ensure comparability. First, the irregular porous structures exhibited porosity levels equal to 70, 80, and 90%. Fifteen kinds of porous structure models with average apertures of 600, 700, 800, 900, and 1,000 μm were constructed. The design parameters are determined by combining the fitting formula of all characteristic and design parameters, as shown in Figure 2:

The solution method of design parameters is based on the function relationship between characteristic parameters and design parameters established above. Before design, the average aperture and porosity of irregular porous structure are determined. Combining all fitting formulas of characteristic parameters and design parameters, the solution method of design parameters can be obtained as follows:

1) The coefficient of irregularity is between 0.4 and 0.5;
2) According to the porosity requirements, plug into *Φ* = 107.87K-1.33 to calculate the corresponding pore size coefficient K;
3) According to the aperture requirements, plug into Eq. 1 to solve the corresponding point spacing a;
4) According to the irregularity coefficient formula *ε* = R/a, the probability sphere radius R is solved;
5) Adopt the Voronoi tessellation structural design method to design the porous structure.

**FIGURE 3:**
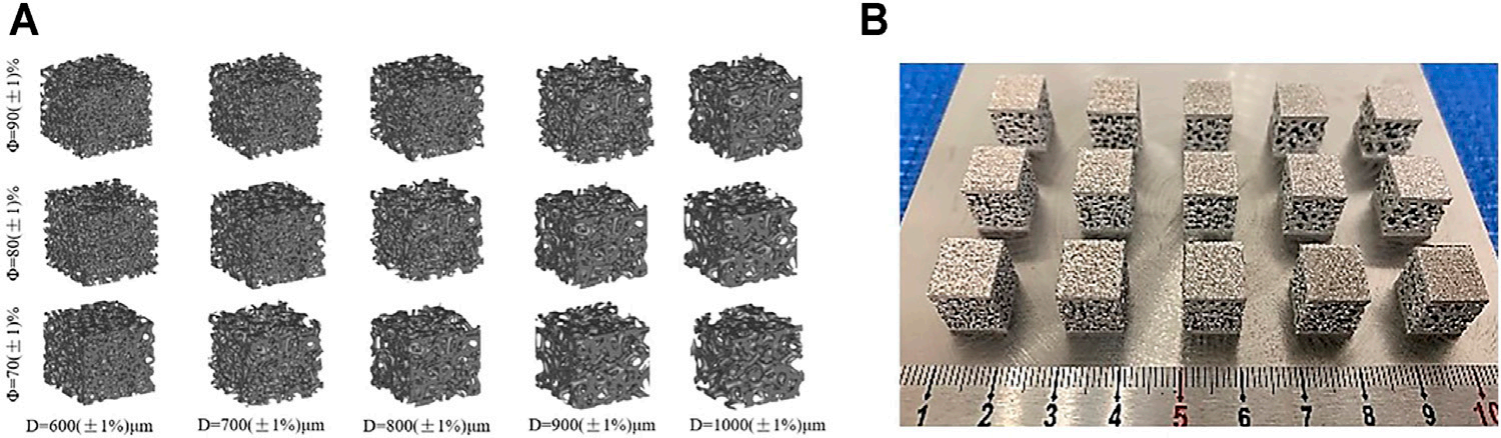
Irregular porous structure model. **(A)** Design model; **(B)** Print sample.

The above method can be used to design three different porosity structures, thereby determining the design parameters, as shown in Table 1. The irregular porous structure model obtained according to the design requirements is shown in Figure 3A, The printed porous structure sample is shown in Figure 3B.

**TABLE 1:** Design parameters of porous structure.

D	*Φ* (%)	K	a	r
600	70	0.66	1.43	0.64
	80	0.75	1.25	0.56
	90	0.84	1.11	0.50
700	70	0.66	1.67	0.76
	80	0.75	1.47	0.66
	90	0.84	1.31	0.59
800	70	0.66	1.91	0.86
	80	0.75	1.69	0.76
	90	0.84	1.51	0.68
900	70	0.66	2.14	0.96
	80	0.75	1.89	0.85
	90	0.84	1.69	0.76
1,000	70	0.66	2.37	1.07
	80	0.75	2.10	0.95
	90	0.84	1.88	0.85

### 2.2. Finite Element Analysis of Mechanical Properties

**FIGURE 4:**
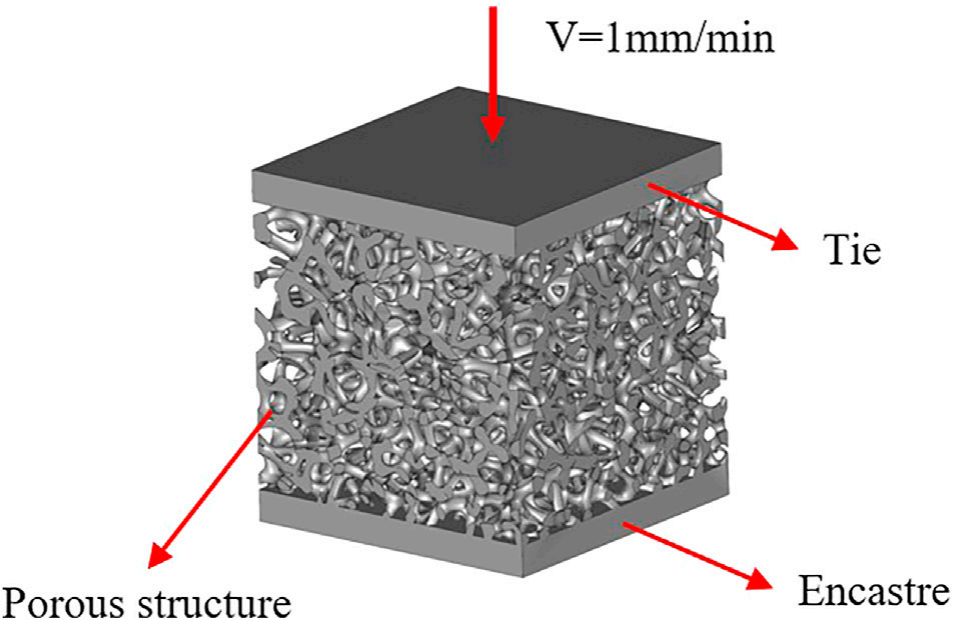
Finite element analysis model.

The study on the mechanical properties of a porous structure currently includes four major aspects: compressive strength, tensile strength, bending strength, and fatigue strength (Cao et al., 2018; Qiu et al., 2018). This study mainly examines the simulation of compression testing, including the effective elastic modulus, maximum compressive strength, and stress distribution, among others. To analyze the influence of structural parameters on the mechanical properties of the designed porous structure, the commercial finite element software ABAQUS was used in the simulation of the structure. First, the model built in Grasshopper needed to be exported in the STL format, imported into the software 3-Matic to generate the volume grid, and finally exported in the INP format. The finite element simulation of the compressive strength of the porous structure is presented in Figure 4. The porous structures of the upper and lower sets of rigid materials, respectively, in the upper model induce a downward movement at a speed of 1 mm/min. The bottom of the model fully controlled, the other in the process of simulation, the simulation environment for the static, general motors, all models according to set the properties of the Ti-6Al-4V material. The density is 4.41 g/cm^3^, the elastic modulus is 110 GPa, and Poisson’s ratio is 0.33.

### 2.2 Compression Test

**FIGURE 5:**
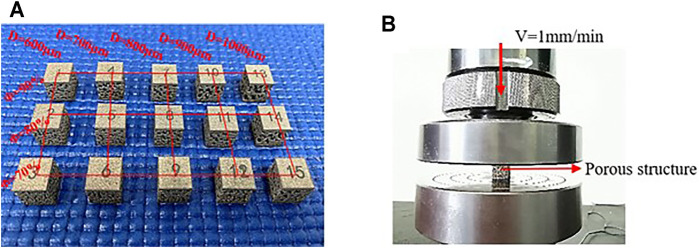
**(A)** Print sample; **(B)** Compression test.

The specimens were fabricated using an SLM machine (NCL-M2120, China) with optimized processing parameters: laser power of 130 w, scanning speed of 1,000 mm/s, and hatch spacing of 0.08 mm. The material used was Ti-6Al-4V powder with a diameter range of 15–53 μm fifteen sets of specimens were fabricated with a height of 12 mm. The prepared porous structure was numbered (Figure 5A) to facilitate the experimental analysis. No support structure was used during processing to ensure the stability of the support. To facilitate the compression test, solid structures were set at the upper and lower ends of the porous structure. In the figure, both the interior and exterior appear precisely manufactured. Compression tests were performed on a mechanical testing machine (CMT5105, MTS Systems, United States). The crosshead displacement velocity was fixed at 1 mm/min. The compression experiment process is presented in Figure 5B.

### 2.4 Analysis of Permeability Characteristics

**FIGURE 6:**
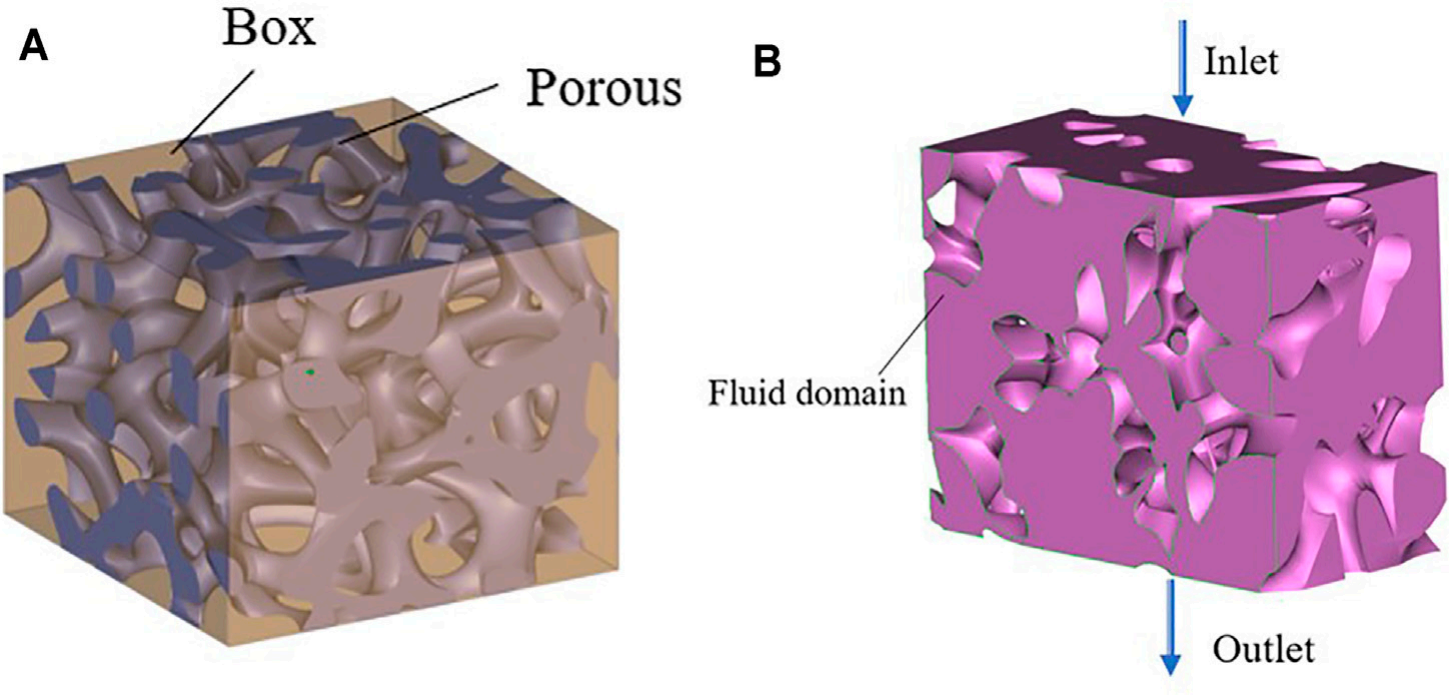
Penetration simulation preparation process. **(A)** Boolean operation; **(B)** Fluid domain model.

Computational fluid dynamics (CFD) modeling was performed using the software COMSOL. The simulation model was first determined after the porous structure was removed *via* a Boolean operation in Grasshopper (Figure 6A). The output was then and saved in the STL format. The software 3-MATIC was used to import the volume mesh components into COMSOL for simulation analysis.

CFD modeling is approximated by single-phase and peristaltic flow models. To simplify the simulation calculation and analysis, the deformation of the metal scaffolds is ignored during the fluid flow process. Water was assigned as the fluid domain material, with the following physical properties: temperature, 37°C (normal human body temperature); density, 1,000 kg/m^3^; and viscosity, 1.45 E^−9^ MPa/s. In CFD modeling, the Reynolds number is typically used to assess the state of the fluid. The analysis object is an incompressible fluid with constant density; thus, it is defined by the Navier–Stokes equation (Wang et al., 2016; Cao et al., 2018), as shown in Eq. 2.

$$\rho \frac{\partial v}{\partial t}=-\left(v\cdot \nabla \right)v-\frac{1}{\rho }\nabla P+\mu \nabla^{2}v+F\nabla \cdot v=0$$ (2)

where *ρ* is the density of the fluid (kg/m^3^), v is the speed of the fluid (m/s), *t* is time (s), ∇ is the operator, *P* is the pressure (MPa), *μ* is the dynamic viscosity coefficient of the fluid, and *F* is the force (N).

The boundary conditions for the fluid model are presented in Figure 6B, with the purple section as the fluid domain, defined as the inlet boundary and the outlet boundary. The inlet velocity applied to the scaffolds was 1 mm/s. The pressure at the exit is considered zero. Under the no-slip-on-the-wall assumption, the fluid flow in the bionic bone scaffolds was simulated using COMSOL.

The results report on the pressure drop, pressure gradient, porosity, outlet flow rate, and the permeability between the inlet and outlet surface of the watershed. The permeability was determined in accordance with Eq. 3 of Darcy’s law, and the pressure gradient was measured using Eq. 4.

$$K=v_{D}\cdot \mu_{d}\cdot \left(\frac{L}{\Delta P_{i-0}}\right)$$ (3)

$$\Delta P=P_{Inlet}-P_{Outlet}$$ (4)

where *K* is the permeability, *V*_*D*_ is the Darcy velocity, *u*_*d*_ is the dynamic velocity, *L* is the model length, and *P* is the pressure gradient of the fluid domain.

### 2.5 Biocellular Culture

The prepared Ti-6Al-4V scaffold was treated before cell culture. First, the scaffold was ultrasonically rinsed for 30 min in 95% alcohol and distilled water, then soak for 24 h in a 5 M sodium hydroxide solution at 60°C, and then ultrasonically washed in distilled water for 10 min and dried for 24 h to stabilize the oxide layer of the Ti-6Al-4V scaffold prior to cell culture. Finally, all scaffold were sterilized using high temperature and high pressure.

Osteoblasts from mice (CELL Bank MC3T3-E1, Chinese Academy of Sciences) were used to evaluate the permeability of the scaffold. The cell culture temperature was set to 37°C, and the environment contained 5% CO_2_. The medium used for cell culture was α-MEM containing 10% fetal bovine serum and 3% penicillin–streptomycin (Gibco). The cells were seeded on sterilized samples at a density of 5 × 10^4^ cells/cm^2^ in a 24-hole plate. After one, three, five and 7 days of culturing, the absorbance was measured at 450 nm using a microplate reader (Multiskan GO, Thermo Scientific, United States). According to a previously described protocol, the cells were stained with 4′,6-diamidino-2-phenylindole (DAPI) for 3 h and then observed under confocal fluorescence microscope (CKX53, Olympus Corporation, Japan).

## 3 Results and Discussion

### 3.1 Analysis of Mechanical Properties

#### 3.1.1 Analysis of Compression Test Results

**FIGURE 7:**
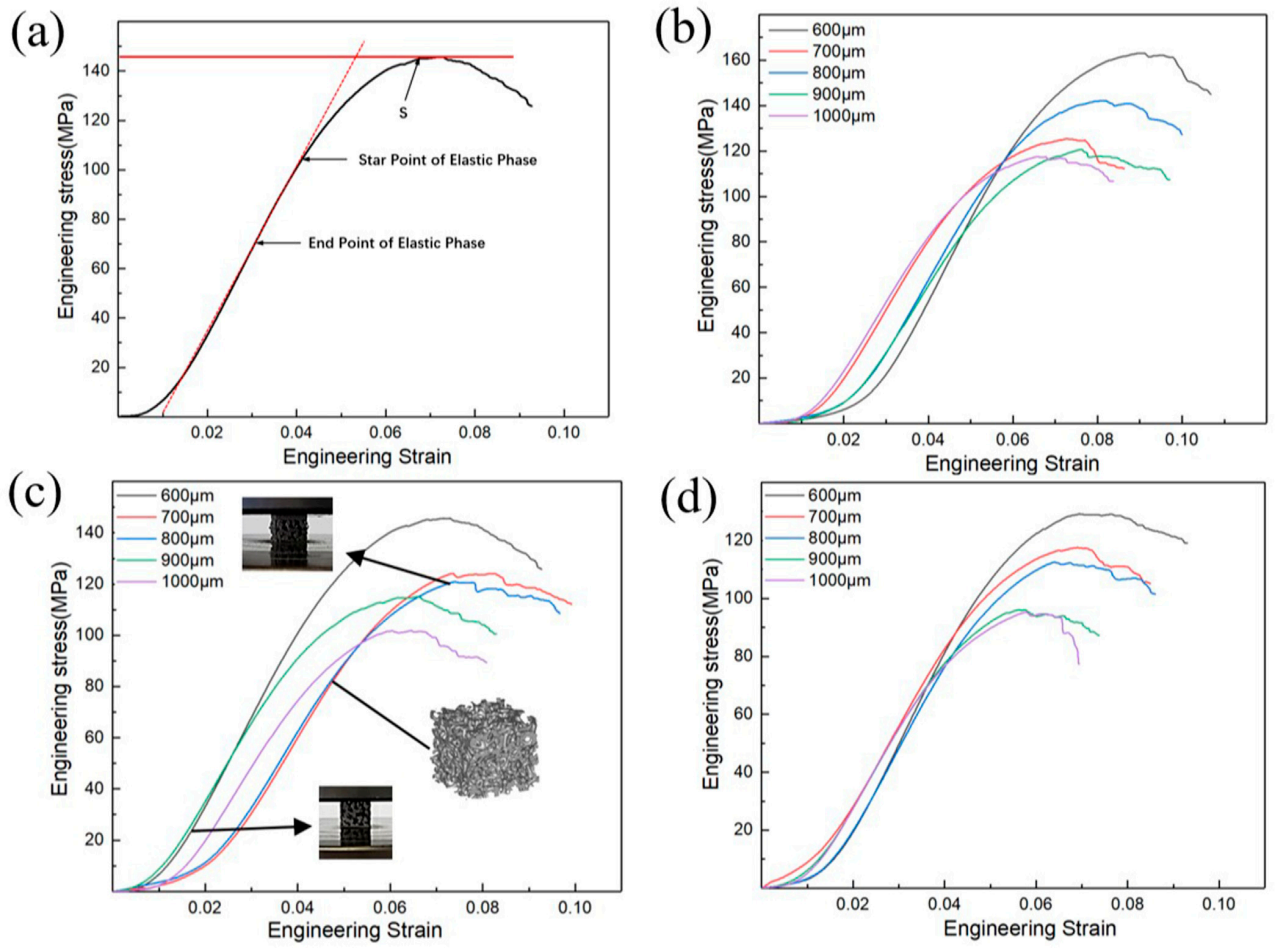
Stress-strain curve of different porosity. **(A)** Engineering stress–strain curve; **(B)** *Φ* = 70%; **(C)** *Φ* = 80%; **(D)** *Φ* = 90%.

In this study, the mechanical properties of porous bone implants include two aspects—the apparent elastic modulus (E) and the ultimate compressive strength (S). E is characterized by the quasi-elastic gradient (ISO13314:2011), and S is characterized by the ultimate compressive stress. The stress–strain curve generated on the testing machine is transformed into the engineering stress–strain curve (Figure 7A). Nonlinearities are observed before the linear elastic phase of the curve. The reason is that full contact is established between the sample and the indenter during compression. The curves also show that the porous structure of titanium alloy exhibits no apparent yield behavior. The compressive strength is characterized by the ultimate compressive stress; the elastic modulus of the sample is the slope of the stress–strain curve at the elastic deformation stage; and the compressive strength is the stress corresponding to the peak of the stress–strain curve.

Mechanical properties are critical evaluation indices for the structure used in orthopedic implants (Wang et al., 2016; Zhang et al., 2018; Cai et al., 2019). The porous structure of the implant can produce a good biological reaction with the host bone *in vivo* and solve the problem of stress shielding only when its mechanical properties meet certain conditions. Moreover, the ultimate strength of the porous structure of the implant should exceed that of the corresponding part of the human bone. Figures 7B–D show porosity levels of 70, 80, and 90%; the quasistatic compression stress–strain curve of the porous structure of a bone implant, and the mechanical deformation processes of the three groups of porous structures (Figure 7C). As shown in the figure, the compression state of the porous structure of the porosity is 80%, and the average aperture is 800 μm. The porous structure fracture occurs at maximum stress. As shown in Figure 7B, when the porosity of the porous structure is 70% and the average aperture is 600 μm, the highest stress is reached; the stress of the porous structure, as determined from the stress curves of (B–D), exceeds the stress range for the natural bone. In addition, some curves exhibit an upward-opening parabola at the start of loading, which is due to insufficient contact between the compression surface and the indenter.

**FIGURE 8:**
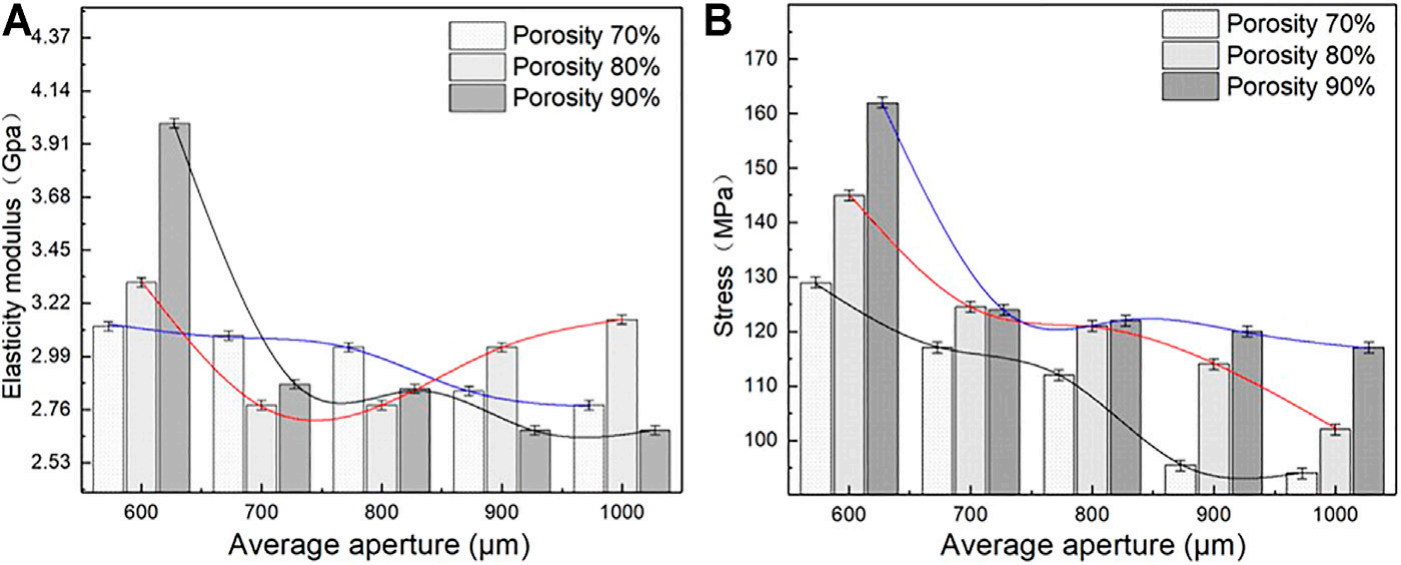
Elastic modulus and Engineering stress of porous structure **(A)** Variation trend of elastic modulus; **(B)** Variation trend of engineering stress.

**FIGURE 9:**
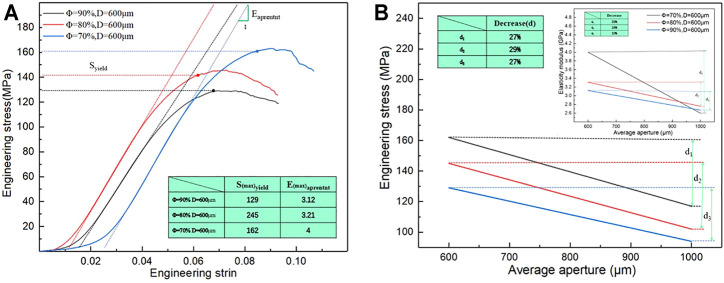
Elastic modulus and engineering stress of difference porous structure. **(A)** Maximum engineering stress curve; **(B)** Variation trend of elastic modulus and engineering stress.

Elastic modulus and compressive strength are two important parameters used to characterize the mechanical properties of porous structures. The matching of the elastic modulus determines whether the structure can solve the stress shielding problem, and the compressive strength determines the maximum load that the structure can bear. Figure 8 lists the elastic modulus and compressive strength of the sample as measured by uniaxial compression testing. As shown in Figures 8A,B, under different porosity levels, the elastic modulus and compressive strength of the porous structure change with the different average aperture. When the porosity is 70%, the elastic modulus and compressive strength of the porous structure tend to decrease with an increase in the average aperture. The elastic modulus and compressive strength are considerably influenced by the average aperture. When the average aperture is 600 μm, The maximum elastic modulus and compressive strength are 4 GPa and 162 MPa, respectively; when the average aperture is 1,000 μm, the minimum elastic modulus and compressive strength are 2.6 Gpa and 117 MPa, respectively, when the porosity is 80%, the elastic modulus of the porous structure initially decreases and then increases with an increase in the average aperture; meanwhile, the compressive strength decreases with an increase in the average aperture. When the average aperture is 1,000 and 600 μm, the elastic modulus reaches the minimum value of 2.76 GPa and the maximum value of 3.31 GPa. When the average aperture is 600 and 1,000 μm, the maximum compressive strength reaches 145 MPa and the minimum compressive strength reaches 102 MPa. When the porosity is 90%, the elastic modulus and compressive strength of the porous structure decrease with an increase in the average aperture. The average aperture slightly affects the elastic modulus, whereas the compressive strength strongly influences the elastic modulus. When the average aperture is 600 μm, the maximum elastic modulus is 3.12 GPa and the maximum compressive strength is 129 MPa. When the average aperture is 1,000 μm, the minimum elastic modulus 2.67 GPa and the minimum compressive strength is 94 MPa. As shown in Figures 9A,B, under the same average aperture, the elastic modulus and compressive strength vary widely as the porosity levels changes. When the average aperture is 600 μm, the elastic modulus and compressive strength reach their maximum values. When the average aperture is 1,000 μm, the elastic modulus and compressive strength reach their minimum values. Given different average apertures, both the elastic modulus and compressive strength are within the range of the bone tissue.

Figure 9B also shows the decreases in elastic modulus and compressive strength with an increase in the average aperture. When the porosity is 70%, the elastic modulus and compressive strength decrease by 33 and 27%, respectively, with a change in the average aperture; when the porosity is 80%, the elastic modulus and compressive strength decrease by 16 and 29%, respectively; when the porosity is 90%, the elastic modulus and compressive strength decrease by 10 and 27%, respectively. Under different porosity conditions, the compressive strength of the porous structure varies within a range similar to that of the average aperture, and the elastic modulus largely affects the average aperture. This finding indicates that the porosity only slightly affects the compressive strength but heavily affects the elastic modulus.

In conclusion, the porous scaffolds prepared using Ti-6Al-4V can not only substantially adjust the elastic modulus via the average aperture but also ensure high compressive strength, which shows significant potential in the application of bone scaffolds.

#### 3.1.2 Mechanical Finite Element Simulation Analysis

**FIGURE 10:**
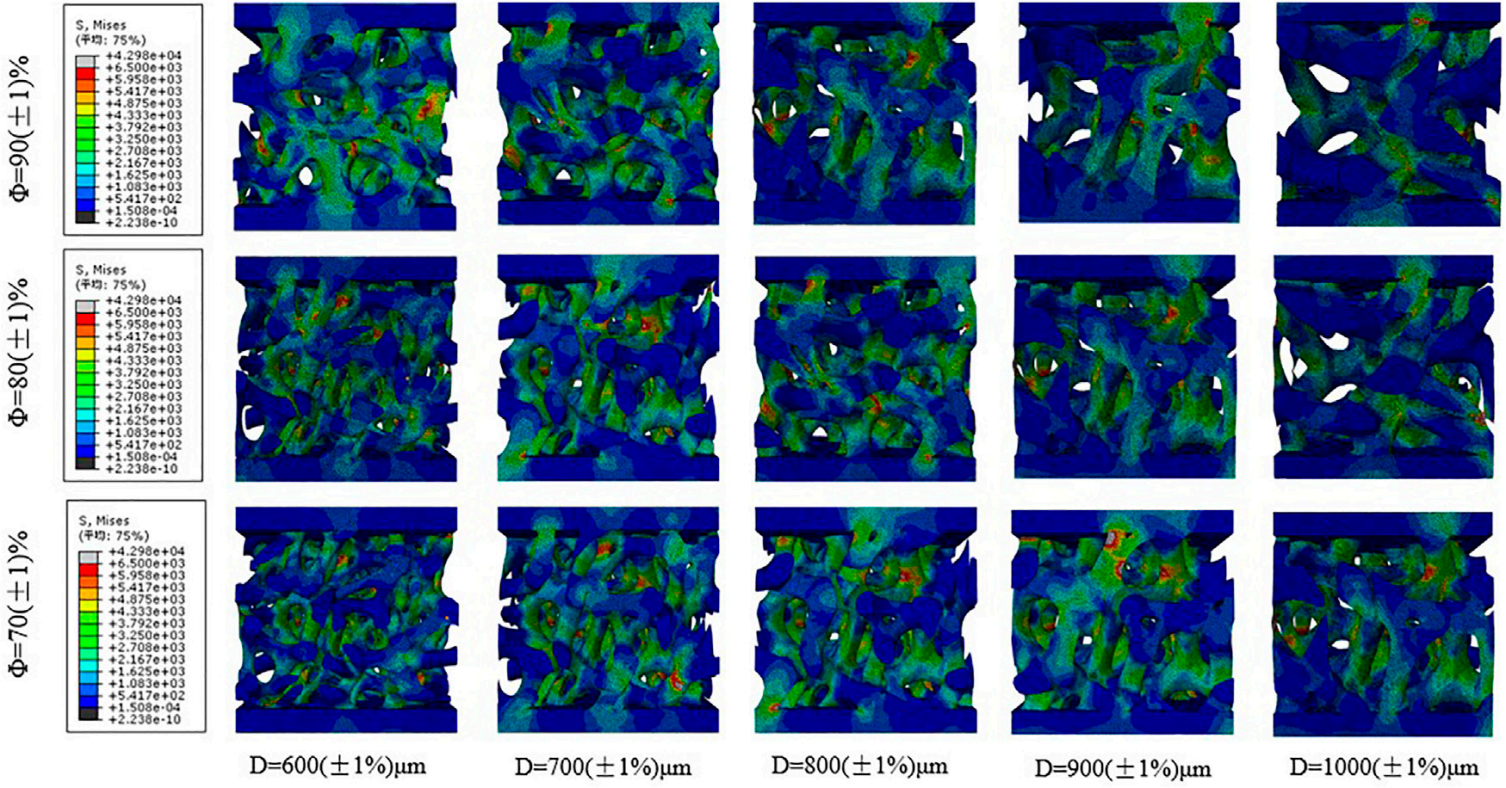
Stress distribution of the scaffolds with different average aperture and porosity under uniformly distributed load.

Figure 10 shows the stress distribution corresponding to the different average apertures of bone implants when the porosity levels are 70, 80, and 90%. As shown in the figure, the maximum Mises stress of the porous structure of bone implants is mainly concentrated at the nodes where the connecting rods of the porous structure are connected, and the randomness of the porous structure facilitates the production of fragile and brittle pore edges. With regard to bearing loads, the stress is often more concentrated on the fragile and brittle pore edges. The force can be gradually transferred via connecting rods from the loading area to the different layers of the layered slice. As shown in Figure 9, the smaller the average aperture, the greater the stress on the pore edge of the porous structure when the porosity level is the same; meanwhile, under different porosity conditions, the loading state of the porous structure is similar when the average aperture is the same.

Therefore, compared with the average aperture, the porosity level exerts less effect on the stress of the porous structure, which is consistent with the results of the mechanical compression test. In the design of porous structures, not only should the porosity of the porous structure be paid attention; the appropriate average aperture also needs to be ensured. Prevention of structural fracture caused by aperture distribution and reasonable control of the average aperture can effectively improve the average stress of an irregular porous structure, as well as enhance the compressive strength of the structure.

### 3.2 Permeability Analysis

#### 3.2.1 Computational Fluid Dynamics Simulation

**FIGURE 11:**
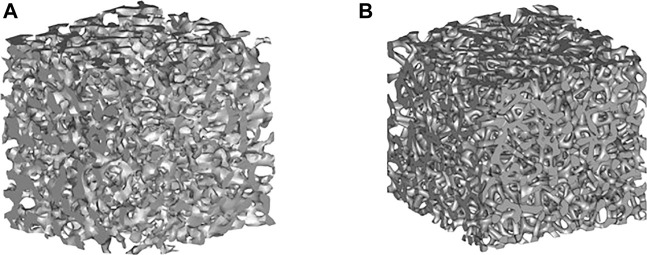
**(A)** Trabecular structure; **(B)** Porous structure.

Material transport is an important index of a biomimetic bone-implant scaffold. Tissue regeneration requires continuous absorption of nutrients via porous channels. Therefore, prediction and evaluation of the permeability and pressure drop of the structure are necessary (Peng et al., 2019; Li et al., 2020; Shuai et al., 2020; Yang et al., 2020; Yang et al., 2021; Zhang et al., 2021). After treatment, the porous structure exhibits a more bionic morphology, as shown in Figure 11. The porous structure similar to the trabecular bone can be simulated by changing the porosity and average aperture of the porous structure.

**FIGURE 12:**
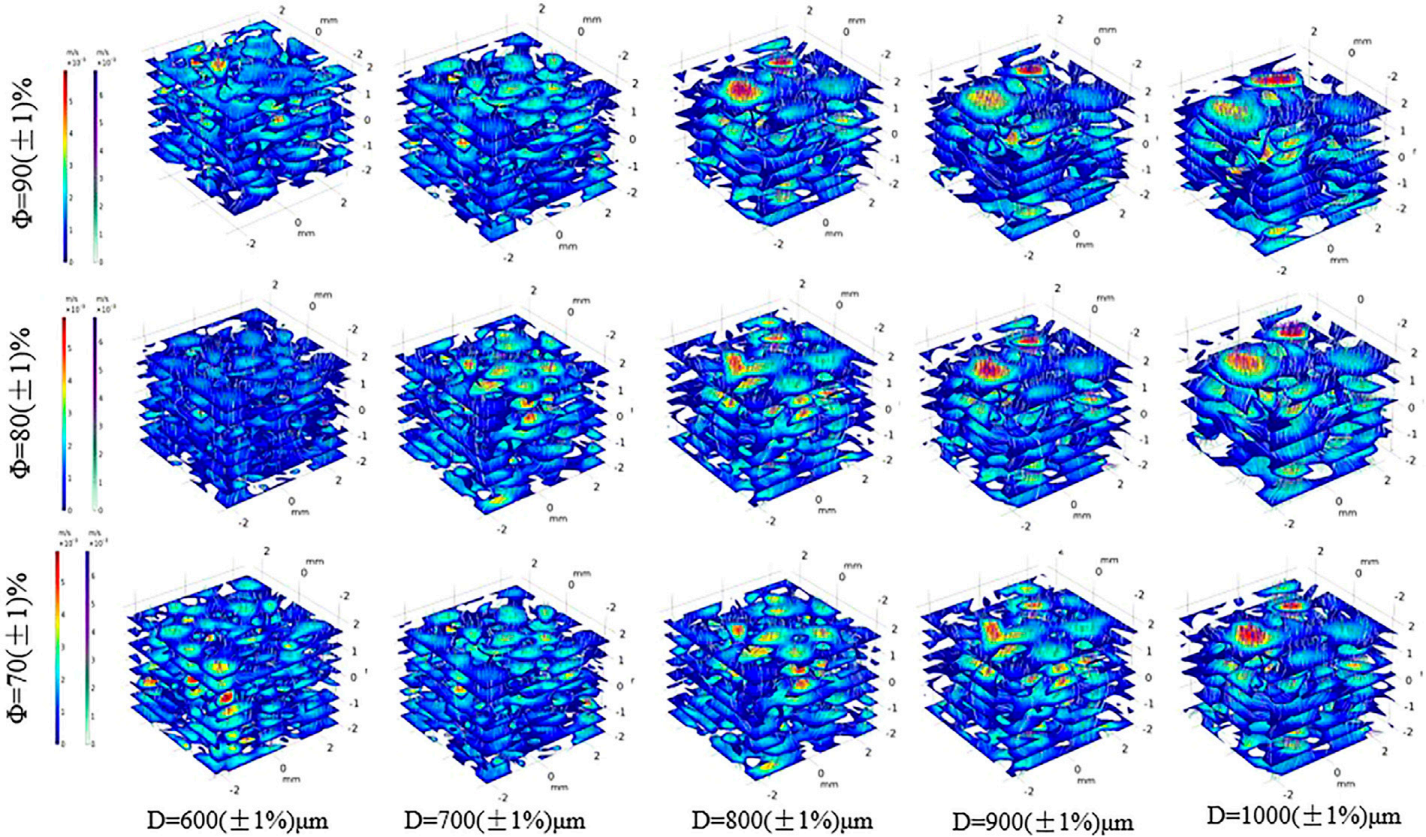
Velocity cloud map distribution of different porous structure.

Fifteen groups of porous structures were analyzed by fluid dynamics simulation. Bone implants possess similar porous structures and vary in the average aperture and porosity of the structures; thus, the pressure drop and permeability of the porous structures need to be evaluated. Pressure drop and permeability are used to quantify the transmission performance between different structures. Figure 12 presents the velocity distribution cloud map corresponding to different average apertures determined via fluid flow simulation at porosity levels equal to 70, 80, and 90%. As shown in the figure, the porous structure of bone implants exhibits a disordered fluid velocity distribution, and the maximum velocity is normally concentrated in the area with a small aperture. Comparison of figures indicates that the velocity distribution varies between different porous structures. Under different average apertures and similar porosity levels, the pressure distribution and velocity distribution are markedly affected by average aperture and slightly influenced by porosity. The larger the average aperture, the faster the nutrient transport and cell attachment; however, a larger-than-average aperture tends to result in weak mechanical properties. The requirements for the mechanical properties of bone scaffolds cannot be satisfied; thus, the mechanical properties of different structures, as well as the cell permeability and pressure drop, should be considered.

**FIGURE 13:**
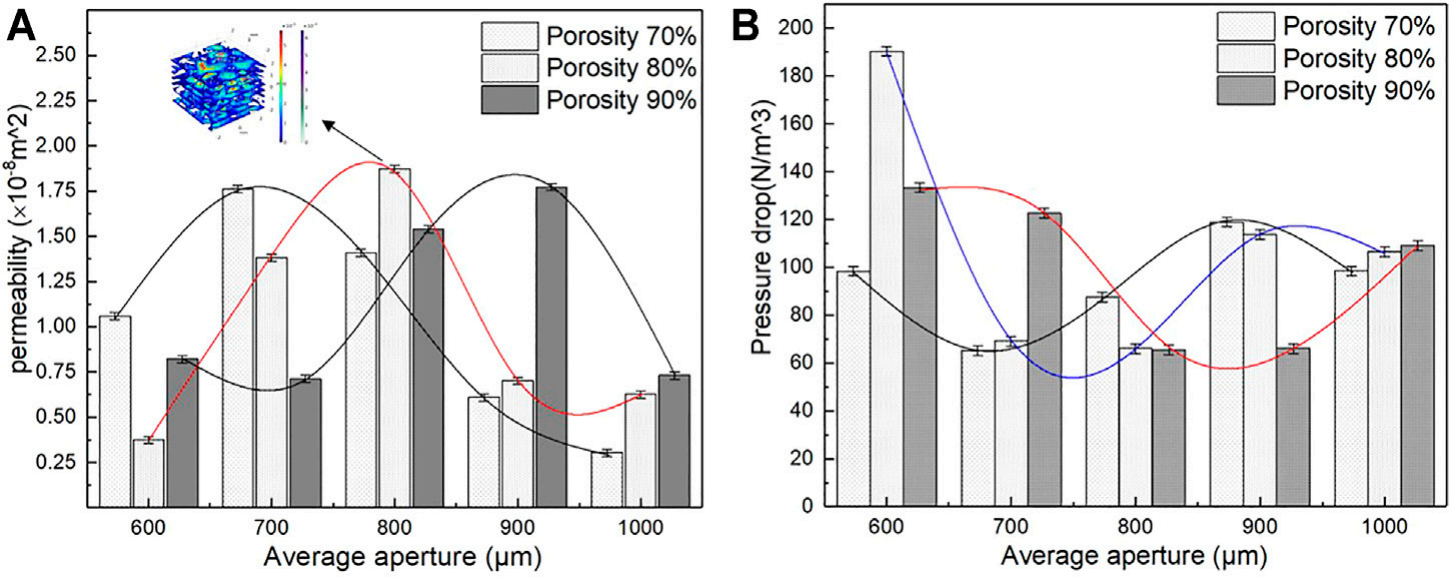
Permeability and pressure drop of different porous structure. **(A)** Variation trend of permeability; **(B)** Variation trend of pressure drop.

Under similar conditions, pressure drop and permeability were calculated using Eqs 3, 4. The results are shown in Figure 13. As indicated in Figure 13B, the pressure drop initially decreases and then rises with an increase in average aperture, and the small average aperture leads to a larger pressure drop and a higher flow rate. When the average aperture is 800 μm and the porosity is 80%, the pressure drop reaches 66 N/m^3^, which is conducive to cell adhesion. The structure with an average aperture exceeding 800 μm exhibits an increasing flow rate, which is not conducive to cell adsorption. As observed in Figure 13A, the permeability of the porous structure under different porosity levels increases first and then decreases with an increase in the average aperture. The main reason is that the average aperture increased, the internal surface area of the model increased, and the fluid friction increased; thus, the velocity was reduced, resulting in a decrease in permeability. When the porosity is 80%, and the average aperture is 800 μm, the maximum permeability reaches 1.87 E^−8^ m^2^, which is a considerably larger value than that of the natural bone. On the basis of the aforementioned data, the permeability of the porous structure of bone implants is greater than that of the human bone. Moreover, the flow characteristics may promote tissue growth, showing great research potential and application prospects.

**FIGURE 14:**
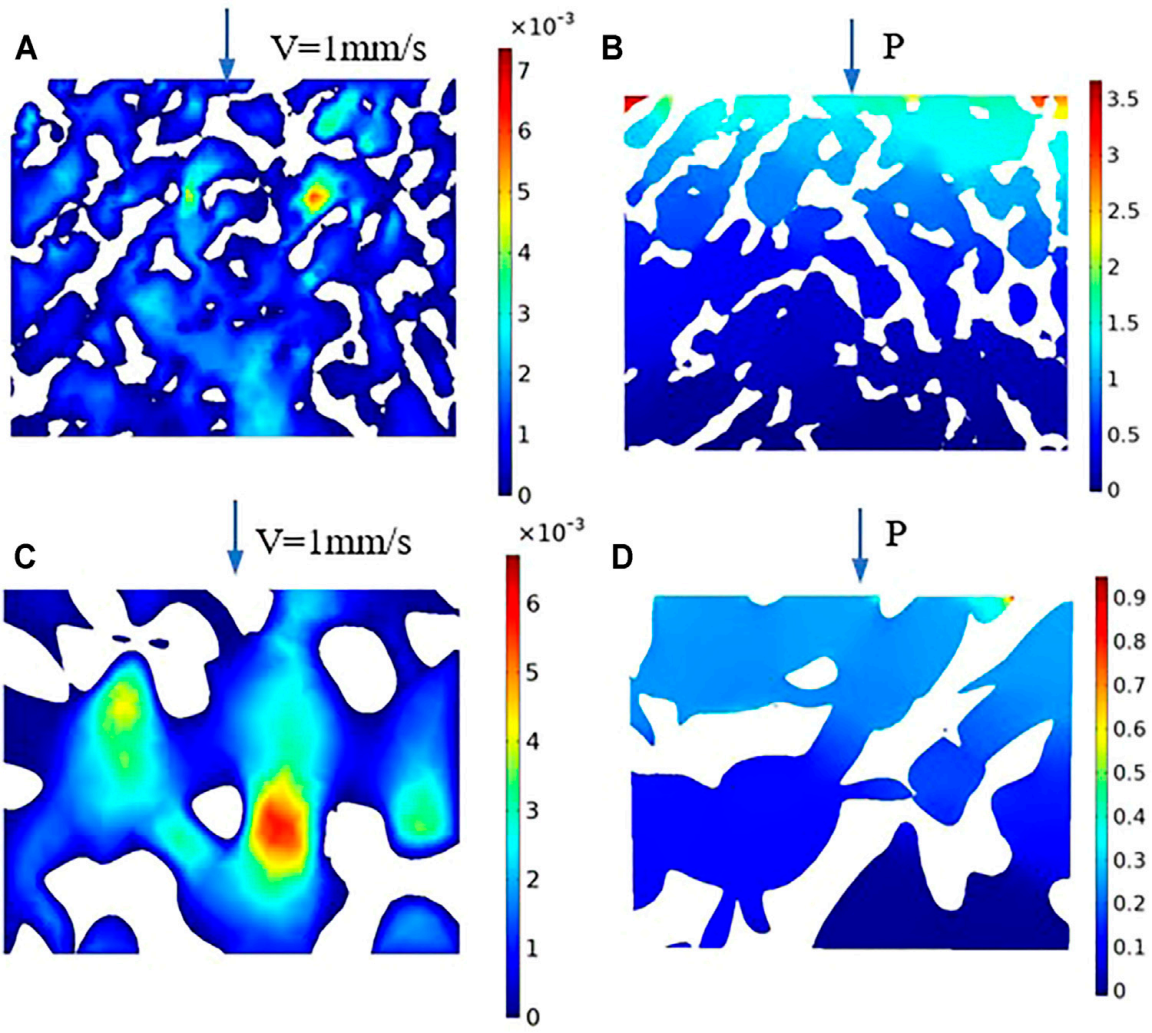
Velocity and pressure section cloud diagram. **(A)** Natural bone velocity distribution cloud map; **(B)** Natural bone pressure cloud map; **(C)** Velocity distribution cloud map of the porosity is 80%; **(D)** Pressure cloud map of the porosity is 80%.

Figure 14A presents the cross-section of the natural bone velocity distribution cloud map, and Figure 14B shows the cross-section of the natural bone pressure cloud map. As shown in the figure, the fluid velocity is disordered, the maximum flow velocity is concentrated in the area with a small aperture, and the pressure gradually decreases from the inlet to the outlet; meanwhile, the pressure is stable in the area with a large aperture. Moreover, the permeability is 1.50 E^−10^ m^2^, and the pressure drop is 4.153 E^3^ N/m^3^. Figures 14C,D present the cross-section of the velocity distribution and pressure distribution when the porosity is 80% and the average aperture is 800 μm. In Figure 14C, the velocity distribution is the highest at the center of the inlet and outlet of the porous structure. Figure 14D shows that the pressure of the porous structure decreases gradually from the inlet to the outlet. Moreover, it decreases in the radial direction from the center to the inner boundary. This flow characteristic can be more intuitively and clearly visualized using the diagram. The high velocity at the center of the implant porous structure facilitates the migration of cells and nutrient materials deep into the scaffold. The closer to the boundary of the inner surface, the lower the velocity, which facilitates the absorption of cells and nutrients on the inner surface of the scaffold. These substances are essential for bone tissue growth and promote subsequent development.

With the special structural characteristics of irregular porous structures considered, not only are their pores randomly distributed in space, their aperture is also distributed within a certain range. Notably, this porous structure has a number of large pores distributed in space, which are less permeable and suitable for cell adhesion and proliferation; moreover, the large number of pores ensures the flow of oxygen and nutrients. Compared with that of the natural bone structure, the controllability of the porous structure design parameters of the bone implant can be controlled by adjusting the porosity and average aperture in the irregular porous scaffold. S. Gomez et al. constructed an irregular porous structure by using the Voronoi surface subdivision method. When the porosity ranged from 40 to 90%, the permeability values range from 0.5 < K (×10^−8^ m^2^). By contrast, the irregular porous structure in the current study had a wider permeability range and greater permeability control. The surface area of the actual porous sample after additive manufacturing tends to be larger than that of the design, and the friction of the liquid flow also increases. With these observations considered, the porous model must have a larger penetration range than that of the aforementioned, and the permeability of the porous structure with different average apertures in the figure can be met.

**FIGURE 15:**
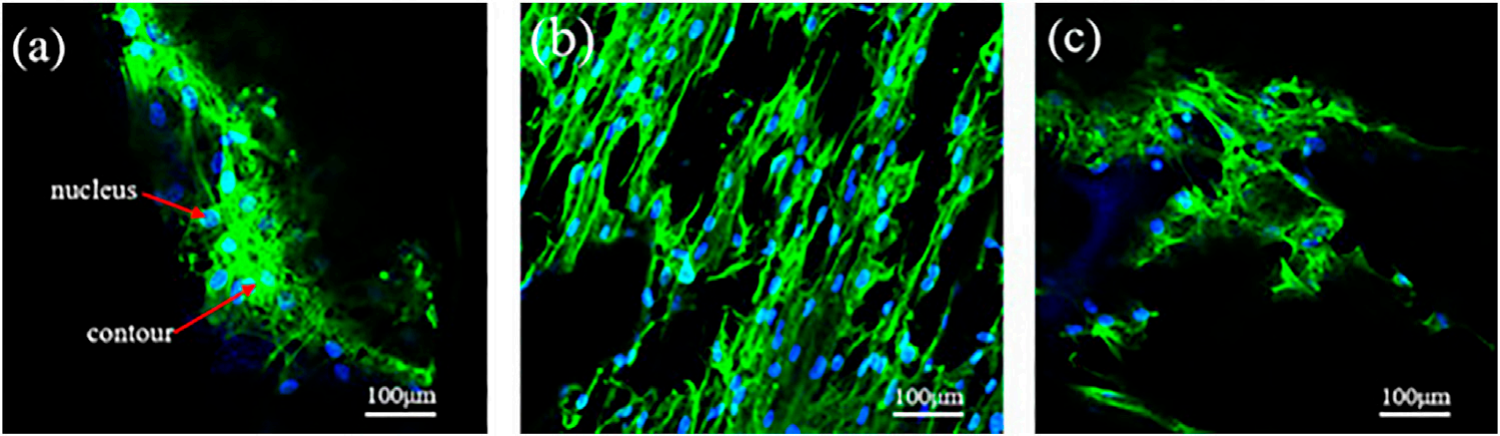
Confocal fluorescence microscope images of MC3T3-E1 cells (indicated by arrows) adhered to the scaffold surface after incubating for 7 days. **(A)** *Φ* = 80%, D = 600 μm; **(B)** *Φ* = 80%, D = 800 μm; **(C)** *Φ* = 80%, D = 1,000 μm.

The average aperture not only affects the mechanical and permeability properties of the porous scaffolds, but also affects the adhesion of bone cells. Mc3t3-e1 cells were inoculated on the scaffold, after culture for 7 days, the cells attach to the walls of the scaffold was observed by confocal fluorescence microscope. In Figure 15, green represents the cell contour, and blue represents the nucleus. Figure 15 shows the confocal fluorescence microscope images of MC3T3-E1 cells (indicated by arrows) attach to the scaffold surface after incubating for 7 days. As known from the analysis of fluid dynamics, a larger average aperture and higher porosity do not indicate a higher permeability. When the average aperture is 800 μm, Figure 15B shows the scaffold distribution of some larger and smaller pores, allowing more cells to be adhered, simultaneously, the maximum permeability of porous scaffolds reaches 1.87 E^−8^ m^2^, this permeability is greater than natural bone, resulting in a higher cell adhesion rate, more cells attach to the walls of the scaffold, and the effect of cell pseudopodia was better. In the same range, when the average aperture is 600 and 1,000 μm, the permeability of porous scaffolds is relatively low, Figures 15A,C shows the cells adhere to the scaffold less than Figure 15B, and the effect of cell pseudopodia was not obvious.

The difference in the cell density of several groups of scaffolds in culture is mainly attributed to the difference in the local permeability of the porous structure, which can directly affect the efficiency of cell adhere to the walls of the scaffold. The permeability of porous scaffolds is related to the porosity and average aperture (Dias et al., 2012; Fan et al., 2013; Torres-Sanchez et al., 2021b); a higher permeability indicates that the cell is subjected to less resistance when it penetrates the scaffold. Consequently, the cells adhere to the scaffold surface for a shortened time. Although the overall porosity and average aperture are roughly similar, the variation in average aperture leads to different permeability levels. The larger average aperture and higher porosity of the scaffolds can facilitate nutrient transport, address the oxygen gap, and prevent congestion; thus, it is beneficial to maintain activity and cell proliferation. A suitable aperture range is the first prerequisite to ensure bone ingrowth, in addition to cell proliferation and differentiation. It is also the most direct and important functional parameter of the porous structure as a medical implant. Extremely large apertures and bone cells cannot adhere, resulting in bone tissue loss and poor mechanical strength. The aperture is too small for bone cells to grow in, and the tissue fluid cannot be transported efficiently.

## 4 Conclusion

The rational structural design of the trabecular-like porous scaffold is an important factor for the satisfactory clinical effect of the implant. Bone tissue is a three-dimensional entity with a heterogeneous structure, indicating that the ideal implant is composed of a layered structure similar to bone tissue on a multidirectional scale. In addition, the implant should have appropriate biological and biomechanical properties similar to those of the host bone and surrounding tissue. The internal pores of most porous scaffolds are identical, in contrast to the structure of real bone. Consequently, the accuracy of the complex geometry and continuity of the implant at a specific location is difficult to ensure.

The elastic modulus of natural bone is 0.1–23 Gpa, and the compressive strength is 1.5–151 MPa, compared with the natural bone, the results showed that the performance index of the porous scaffolds is within and superior to the range of the natural bone, the elastic modulus of the porous structure is 2.6–4 Gpa and the compressive strength is 94–162 MPa. The results reveal that the mechanical and permeability properties of the porous scaffolds of bone implants designed using the Voronoi–Tessellation method can directly depend on the porous structure of the microstructure characteristics. The simulation results are verified by the biological cell culture experiment, which shows that the irregular porous scaffolds have a wider and more uniform pore size distribution. The combination of small and large pores achieved satisfactory cell attachment efficiency. In future studies, the cell experiment will further validate the best design parameters, and the animal implantation experiments based on the existing research will be conducted to verify the biological characteristics of porous structures.

## Data Availability

The raw data supporting the conclusion of this article will be made available by the authors, without undue reservation.
